# Radiologic–pathologic discrepancy in the tumor size assessment of pancreatic ductal adenocarcinoma in the era of preoperative therapy

**DOI:** 10.1007/s00595-026-03419-5

**Published:** 2026-07-20

**Authors:** Kazuki Kobayashi, Kaya Ijiri, Takazumi Tsunenari, Naoto Yonamine, Mikiya Takao, Takahiro Einama, Hironori Tsujimoto, Hideki Ueno, Sho Ogata, Yoji Kishi

**Affiliations:** 1 Department of Surgery, National Defense Medical College, 3-2 Namiki, Tokorozawa, 359-8513 Saitama Japan; 2 Department of Laboratory Medicine, National Defense Medical College Hospital, Tokorozawa, Saitama Japan; 3 Department of Pathology and Laboratory Medicine, National Defense Medical College, Tokorozawa, Saitama Japan

**Keywords:** Pancreatic ductal adenocarcinoma, Radiologic tumor size, Pathologic tumor size

## Abstract

Tumor size plays a central role in the staging, resectability assessment, and response evaluation of pancreatic ductal adenocarcinoma (PDAC). However, radiologic tumor size does not necessarily correspond to pathologic tumor size, and this discrepancy has become more clinically relevant in the preoperative therapy era. PDAC is characterized by infiltrative growth, abundant desmoplastic stroma, and ill-defined tumor borders, all of which complicate accurate size assessment on imaging. After preoperative therapy, fibrosis, necrosis, and scattered residual tumor cells further obscure the relationship between radiologic abnormalities and viable tumor burden. This review summarizes the mechanisms underlying radiologic–pathologic discrepancies in tumor size assessment of PDAC, with a focus on computed tomography, magnetic resonance imaging, ultrasound, and pathologic evaluation after preoperative therapy. We also discuss the prognostic implications of imaging-based and pathological tumor sizes and highlight the limitations of size-based response assessment. Finally, we review emerging approaches beyond conventional size metrics, including functional imaging, radiomics, and pathological standardization. Recognizing the biological and treatment-related basis of tumor size discrepancy is essential for improving tumor assessment and refining staging strategies in contemporary PDAC management.

## Introduction

The treatment strategies for pancreatic ductal adenocarcinoma (PDAC) have recently shifted toward a more selective approach, in which surgical indications are determined based on both tumor- and patient-related factors [1]. Owing to the increasing adoption of preoperative therapy, including preoperative chemotherapy and chemoradiotherapy [2], preoperative treatment has been actively implemented even in patients with resectable pancreatic cancer [3], and surgical candidacy is increasingly determined based on the tumor response to treatment. Although these preoperative therapy–centered treatment strategies have demonstrated certain benefits, such as improvement in local disease control and suppression of micrometastatic disease [2, 3], they have also introduced new challenges in the interpretation of conventional imaging assessments.

Tumor size has long played a central role in PDAC management, serving as a key parameter for disease staging [4, 5], resectability assessment [6], and the treatment response evaluation [7]. It has also been reported to be associated with the prognosis [8]. Notably, the tumor category of the tumor–node–metastasis classification defined by the ninth edition of the Union for International Cancer Control [4] is based exclusively on tumor size. However, accumulating evidence indicates that radiologic tumor size does not necessarily correspond to pathologic tumor size, leading to discrepancies between clinical and pathologic staging [9–11]. This discordance persists in the era of preoperative therapies. Radiologic and pathologic tumor sizes do not invariably reflect the same tumor characteristics, and a lack of appreciation of this distinction may result in an underestimation or overestimation of the therapeutic effects of preoperative treatment.

In the current study, we focused on tumor size assessment in PDAC in the era of preoperative therapy and systematically examined tumor size in terms of viable tumor burden, treatment-induced fibrosis, and residual disease patterns from both radiologic and pathological perspectives. Furthermore, we summarize the clinical implications of radiologic–pathologic discrepancies in tumor size assessment with respect to prognostic evaluation and discuss future directions for tumor assessment beyond conventional size-based metrics.

## Challenges inherent in the tumor size assessment of PDAC

### General overview: baseline challenges in treatment-naïve PDAC

Tumor size is a fundamental component of cancer staging systems; however, its conceptual basis differs among the tumor types. In hollow organs, such as the colorectum and esophagus, tumor depth of invasion is the principal determinant of the tumor category [12]. In solid organs, such as the liver and pancreas, tumor diameter is commonly used to define local tumor extent and stage [4, 5].

For many solid-organ malignancies, such as hepatocellular carcinoma, tumors are typically solid and expansile, with relatively well-defined margins on both imaging and gross pathologies [13, 14]. In these settings, radiologic and pathologic measurements of tumor size are generally concordant, and the maximum tumor diameter serves as a reproducible staging parameter [15].

**Fig. 1:**
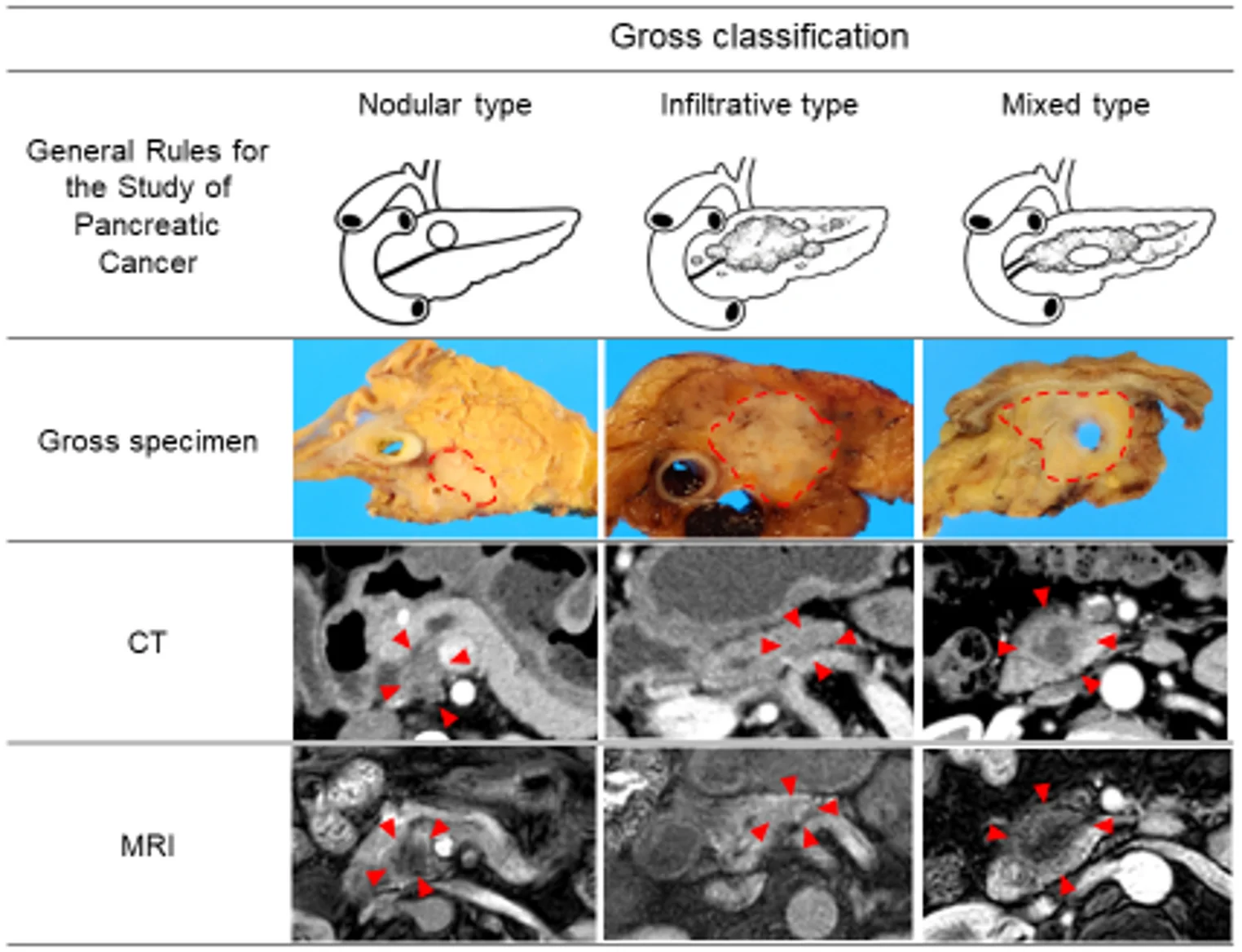
Selected representative gross subtypes of pancreatic ductal adenocarcinoma according to the classification in the *General Rules for the Study of Pancreatic Cancer* of the Japanese Pancreas Society, with corresponding schematic illustrations, gross specimen photographs, CT images, and MRI scans. The tumor areas on CT and MRI are indicated by red arrowheads. The red dashed lines in the gross specimen indicate regions in which tumor cells were microscopically identified

In contrast, PDAC is characterized by an infiltrative growth pattern with abundant desmoplastic stroma and often results in ill-defined tumor borders [16]. Based on this biology, the Japanese Pancreas Society has defined gross tumor classification in the *General Rules for the Study of Pancreatic Cancer* (Fig. 1) [5]. Consequently, clearly delineating tumor boundaries in pancreatic cancer is inherently challenging, complicating the measurement of tumor size. These baseline difficulties are not merely technical issues related to image acquisition. Rather, they reflect a structural mismatch between the region visible as a discrete mass on imaging and the area defined as the tumor extent on pathological examination.

The following sections review how this general problem manifests in each assessment tool, including CT, MRI, US-based techniques, and pathological examinations.

### Computed tomography (CT)–based tumor size assessment

Pancreatic protocol contrast-enhanced CT remains the most widely used modality for tumor size assessment and local staging in PDAC [6, 17]. Current radiologic practice generally defines tumor size as the maximum axial diameter of a hypoattenuating lesion on pancreatic parenchymal or portal venous phase imaging [18, 19]. However, radiologic–pathologic correlation studies have shown that CT-based measurements frequently underestimate the true tumor extent compared with that of pathologic specimens [9–11]. In resected PDAC cohorts, radiologic–pathologic correlation studies have consistently demonstrated that pathologic tumor size exceeds CT-measured size in approximately 80%–85% of patients, with median absolute differences generally in the range of 4–7 mm and relative underestimation exceeding 25% in roughly half of contemporary surgical cohorts [9–11].

This underestimation may result in several patterns of T-stage migration, rather than a single pathway. In our previous study, among patients who did not receive preoperative chemotherapy, cT1 tumors were upstaged to pT2, pT3, and pT4 in 35/55 (63.6%), 11/55 (20.0%), and 5/55 (9.1%) cases, respectively, whereas cT2 tumors were upstaged to pT3 and pT4 in 11/68 (16.2%) and 11/68 (16.2%) cases, respectively [10].

**Fig. 2:**
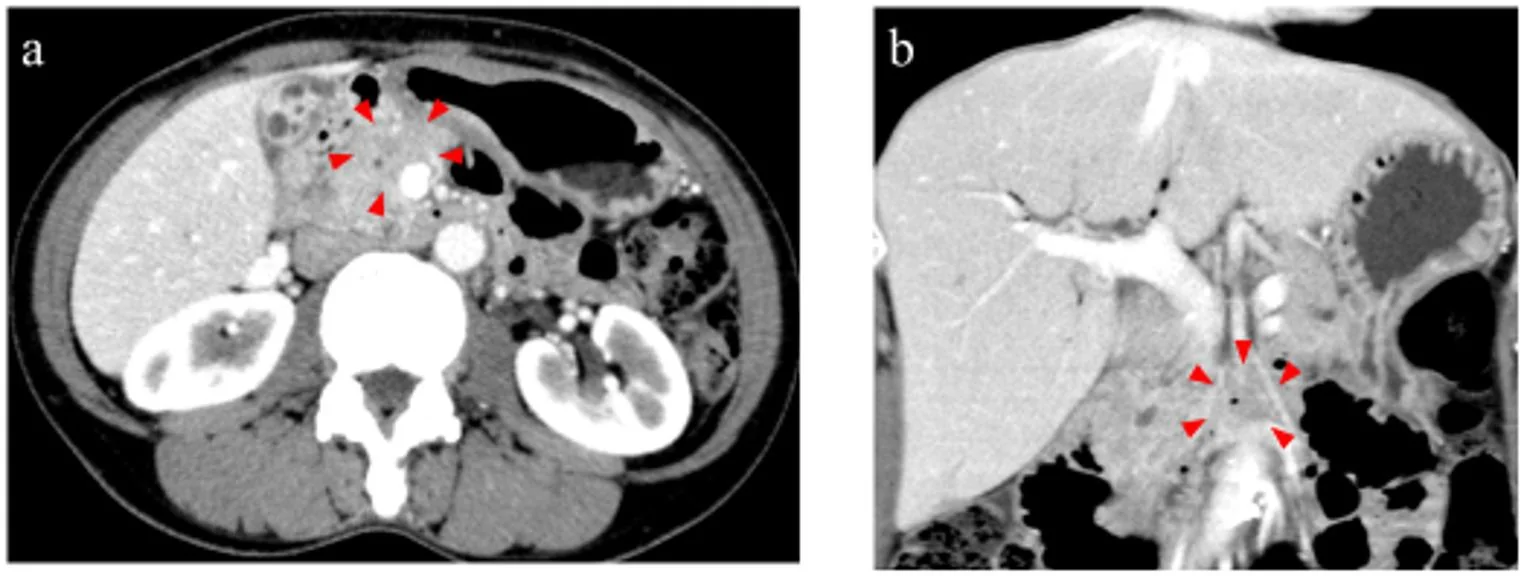
Late arterial phase of contrast-enhanced CT. a: axial view; b: coronal view. An ill-defined hypovascular mass is identified in the pancreatic body (red arrowheads). The radiologic tumor size was 35 mm; however, a pathological examination demonstrated anterior and posterior surface invasions, thus resulting in a pathological tumor size of 65 mm

This discrepancy reflects the biological characteristics of PDAC and the technical and conceptual limitations of CT-based measurements. PDAC exhibits a highly infiltrative growth pattern characterized by discontinuous microscopic spread beyond radiologically visible masses [20–26]. Tumor cells frequently extend along the pancreatic ducts, neural plexuses, and perivascular planes, forming microscopic foci that do not generate a distinct hypoattenuating signal on CT (Fig. 2) [20–24]. These features limit the ability of CT to depict the full microscopic extent of the PDAC.

The desmoplastic stroma that characterizes pancreatic cancer further contributes to this discrepancy. The abundant fibrotic reaction, which consists of a dense extracellular matrix and cancer-associated fibroblasts, creates a hypovascular and heterogeneous tumor microenvironment with a relatively low cellular density [25–28]. This stromal predominance reduces the tumor-to-parenchyma contrast and obscures the tumor margins, particularly at the infiltrative periphery, where viable tumor cells may be interspersed with fibrosis and appear radiologically indistinct from the surrounding pancreatic tissue [9, 16, 17, 26, 27]. Consequently, the pathologic definition of tumor extent does not directly correspond to the radiologic concept of a “visible mass” defined by hypoattenuation on CT.

**Fig. 3:**
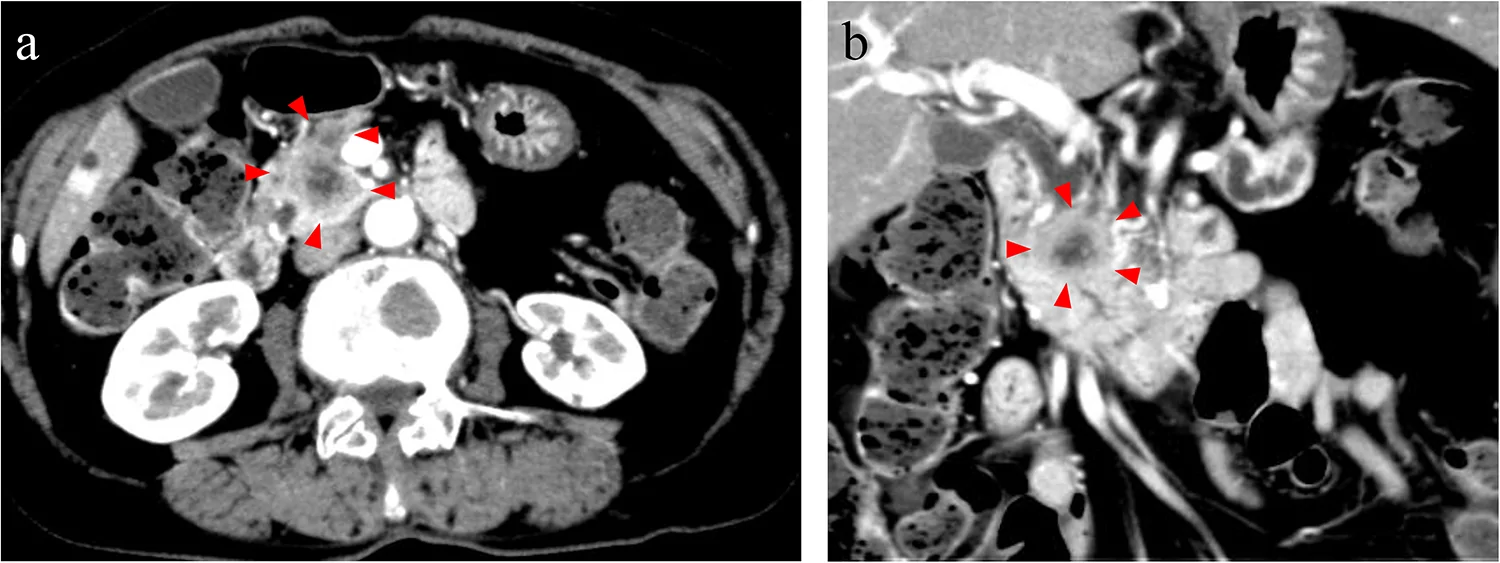
Late arterial phase of contrast-enhanced CT. a: axial view; b: coronal view. A hypovascular mass is observed in the pancreatic head (red arrowheads). Given the anatomic orientation of the pancreas, the maximal tumor dimension was not captured on the CT imaging plane. The radiologic and pathologic tumor sizes were 24 and 33 mm, respectively

In addition to these biological factors, the conceptual limitations of CT-based linear measurements contribute to radiologic–pathologic discordance. Tumor size on CT is typically assessed using the two-dimensional maximum axial diameter, which is inherently sensitive to imaging phase selection, window settings, and slice orientation [18, 19]. Irregular three-dimensional tumor spread, a common feature of pancreatic cancer, cannot be adequately represented by a single, linear metric (Fig. 3). In our previous study, clinical tumor stage (cT)–pathologic tumor stage (pT) concordance was limited to 41%; furthermore, among clinically diagnosed T1 tumors, exact stage agreement (cT1 = pT1) was achieved in only 15% of cases [10].

Interobserver variability is also an important factor in CT-based tumor size assessment. Measurement discrepancies arise from differences in imaging phase selection, window settings, and subjective tumor boundary interpretations [9, 19, 29]. Nevertheless, the reported interobserver agreement is generally good to excellent, with intraclass correlation coefficients (ICCs) ranging from approximately 0.86 to 0.95 [19]. However, such high reproducibility primarily indicates consistency in the application of radiologic definitions and does not necessarily imply an accurate representation of the true pathologic extent of the tumor.

Collectively, the existing literature suggests that CT-based tumor size measurement provides a pragmatic but biologically imperfect surrogate for tumor burden in pancreatic cancer.

### Magnetic resonance imaging (MRI)–based tumor size assessment

MRI is generally regarded as a complementary modality rather than a substitute for CT in tumor size assessment and local staging of PDAC [17, 28]. Although CT remains the standard for radiologic tumor classification, MRI is mainly used when CT findings are equivocal, such as in iso-attenuating tumors or during response assessment [17, 28].

Diffusion-weighted imaging (DWI) improves the conspicuities of lesions. However, diffusion-based parameters, including apparent diffusion coefficient (ADC) values, primarily reflect tissue cellularity and microstructural changes rather than the macroscopic tumor extent [30, 31]. Accordingly, no standardized method exists for defining tumor boundaries or linear tumor sizes based on DWI or ADC measurements.

Contrast-enhanced MRI and pancreatic-protocol CT generally yield comparable linear measurements of tumor sizes. Direct comparative studies have demonstrated small mean differences and high intermodality agreement (ICC ≈ 0.9) between contrast-enhanced CT and MRI [9]. In line with this, another contemporary imaging study reported excellent agreement in measured tumor diameters between CT and MRI, with mean differences of approximately 1.5 mm and ICCs ranging from 0.87 to 0.93 [19, 32].

Notably, even when CT and MRI are concordant, both modalities may still deviate from the pathologic tumor size because both tend to underestimate the true tumor extent on average [9–11, 19]. Therefore, although MRI provides valuable qualitative and biological information, it does not overcome the fundamental limitations of size-based assessments and currently serves as an adjunct, rather than a definitive alternative, to CT.

### Ultrasound (US)–based tumor size assessment

US plays an important but fundamentally supportive role in PDAC tumor size estimation. Transabdominal US is widely used as an initial diagnostic modality, particularly in patients with jaundice. However, pancreatic visualization is frequently limited by bowel gas, obesity, and operator dependency, resulting in inconsistent reliability for linear tumor size assessment in routine practice. Importantly, tumor conspicuity and measurability vary substantially by tumor location in the pancreas, and lesions in the pancreatic tail are particularly difficult to detect on transabdominal US [33–35]. In a study evaluating focal pancreatic lesions detected using endoscopic US (EUS), the detection rates using transabdominal US were 63.6%, 75.8%, and 33.8% for uncinate/head, neck/body, and tail lesions, respectively [35]. Consequently, linear tumor size estimation using transabdominal US may be unreliable in a location-dependent manner, particularly for pancreatic tail lesions.

Even with contrast-enhanced US (CEUS), the accurate delineation of tumor margins remains anatomically constrained and technique-dependent. Prior surgical series focusing on pancreatic head cancer have demonstrated that CEUS yields systematically smaller tumor size estimates than multidetector CT, thus highlighting that US-based linear measurements can vary by both imaging technique and tumor location and may not directly resolve radiologic–pathologic size discrepancies [33].

In contrast, EUS provides high-resolution, close-range imaging of the pancreas and is generally more sensitive than transabdominal US for detecting small pancreatic masses [36]. EUS-related techniques, including contrast-enhanced harmonic EUS, may complement lesion characterization [36, 37]. Accordingly, EUS is routinely incorporated into the diagnostic workup of pancreatic cancer, thus enabling tissue acquisition and pragmatic measurement of the maximal tumor size. In a large clinicopathological study of treatment-naïve pancreatic cancer, preoperative EUS tumor size showed a moderate correlation with postoperative pathological size (*r* ≈ 0.73); nevertheless, pathological tumor size exceeded EUS measurements in approximately 70% of cases, with a median difference of approximately 4 mm, thus suggesting a consistent tendency toward underestimation by EUS [38].

Given that US-based techniques are inherently operator-dependent, measurement reproducibility is an additional consideration. An interobserver and intraobserver agreement study evaluating the EUS-based measurement of primary pancreatic cancer demonstrated good-to-excellent agreement for maximal tumor diameter, with ICC ranging from approximately 0.65 to 0.84 across observers with different experience levels [39]. Although these findings support the technical reproducibility of EUS in selected contexts, they do not overcome the fundamental limitation shared across imaging modalities: the linear tumor diameter remains an imperfect surrogate for the true pathologic tumor extent.

### Pathologic assessment of tumor size

The assessment of pathological tumor size in PDAC is influenced by multiple factors related to specimen handling and processing. Specimen shrinkage during fixation and tissue processing is a well-recognized phenomenon that may affect the measured tumor dimensions, particularly when the tumor size is defined using fixed rather than fresh specimens [40].

In addition, the direction of sectioning and grossing strategies substantially influenced the measured maximal tumor size. Although standardized protocols using axial slicing and multicolor inking have improved the reproducibility of margin assessment and anatomic correlation with imaging, the apparent “maximum tumor size” remains dependent on the slicing plane, slice thickness, and the mapping approach. Consequently, tumor dimensions reconstructed from histologic sections may vary even within the standardized workflows [41–43].

Therefore, the maximum gross tumor diameter does not always reflect the extent of viable tumors, particularly in the presence of extensive fibrosis or inflammation. The additional challenges introduced by preoperative therapy, including treatment-induced fibrosis, necrosis, and discontinuous residual viable tumor foci, are discussed in Chap. 3.

### Summary of the radiologic and pathologic tools

Each radiologic and pathologic tool provides distinct but complementary information for tumor size assessment in PDAC.

- CT remains the standard modality for staging and surgical planning; however, CT-based linear diameter is limited by hypoattenuation-based visibility, imaging plane selection, and the inability to capture microscopic infiltration.
- MRI may improve lesion characterization and provide functional information; however, its linear size measurements are generally concordant with those of CT and therefore share similar limitations relative to pathologic tumor extent.
- Transabdominal US is useful as an initial diagnostic tool, but it is affected by tumor location, bowel gas, body habitus, and operator dependence.
- Endoscopic US provides high-resolution imaging and enables tissue acquisition, although its measurements tend to underestimate the pathological tumor size.
- Pathological examination remains the reference standard; however, the measured tumor size depends on specimen processing, sectioning, sampling, and the operational definition of tumor extent.

These modality-specific strengths and limitations, together with the available quantitative data, are summarized in Table 1.

**Table 1:** Comparison of imaging modalities for tumor size assessment

Modality	Key strengths	Key limitations	Measurement accuracy	Quantitative findings
CT	Standard for tumor size assessment and local staging Basis of radiologic TNM classification	Underestimates true tumor extent Microscopic spread beyond visible mass 2D diameter insufficient for 3D tumor spread	Pathology > CT in 80%–85% Median absolute difference, 5–10 mm > 25% underestimation in ~ 50% of cohorts	cT–pT discordance, 40%–70% Upstaging from lower cT categories, including cT1 → pT2/pT3/pT4 and cT2 → pT3/pT4 High interobserver reproducibility: ICC, 0.86–0.95
MRI	Useful when CT is equivocal Improved lesion conspicuity and DWI-based information	DWI/ADC reflect cellularity, not tumor extent Linear size definition not standardized Size-based limitations remain	Mean CT–MRI size difference, ~ 1.5 mm	CT–MRI agreement: ICC, 0.87–0.93 MRI-specific cT-pT migration data limited
US/CEUS/EUS	Widely available initial modality EUS enables close-range imaging and tissue acquisition	Limited by bowel gas, obesity, and operator dependency Location-dependent visibility Linear diameter remains a surrogate marker	EUS underestimates pathology in ~ 70% Median EUS–pathology difference, ~ 4 mm	EUS reproducibility: ICC, 0.65–0.84 US/EUS-specific cT-pT migration data limited

cT: clinical tumor stage; pT: pathologic tumor stage; CT: computed tomography; MRI: magnetic resonance imaging; US: ultrasound; CEUS: contrast-enhanced ultrasound; EUS: endoscopic ultrasound; TNM: tumor–node–metastasis; ICC: intraclass correlation coefficient; DWI: diffusion-weighted imaging; ADC: apparent diffusion coefficient

## Effect of preoperative therapy on radiologic–pathologic discrepancy

### Mechanisms by which preoperative therapy amplifies underestimation

**Fig. 4:**
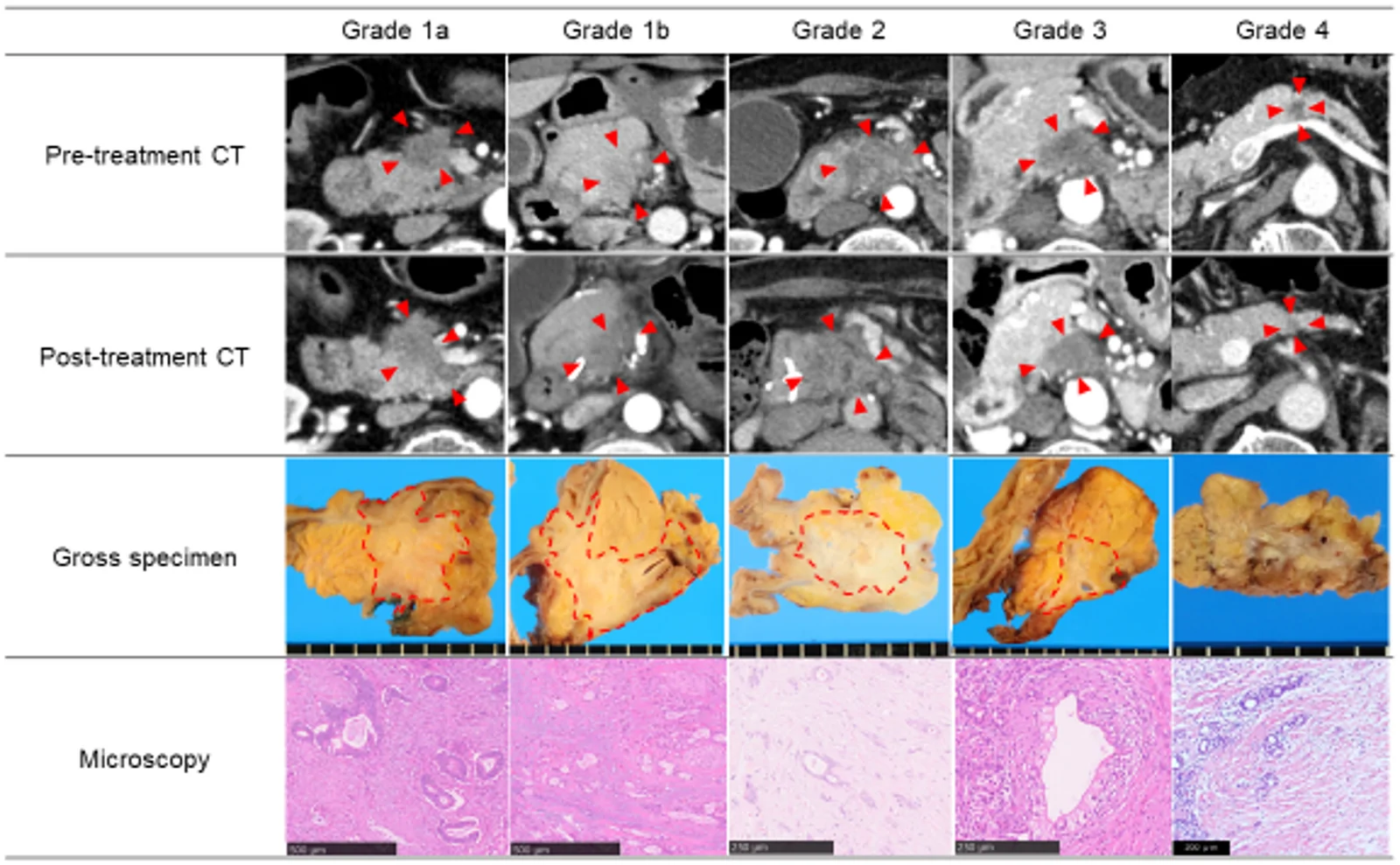
Radiologic and pathologic findings according to the histopathological response grades to preoperative chemotherapy based on the definition in the *General Rules for the Study of Pancreatic Cancer* of the Japanese Pancreas Society. Columns represent the histopathological response grades (Grades 1a–4), and rows show pre-treatment CT, post-treatment CT, gross findings, and corresponding histologic features. The tumor areas on CT and MRI are indicated by red arrowheads. The red dashed lines in the gross specimen indicate regions in which tumor cells were microscopically identified

**Fig. 5:**
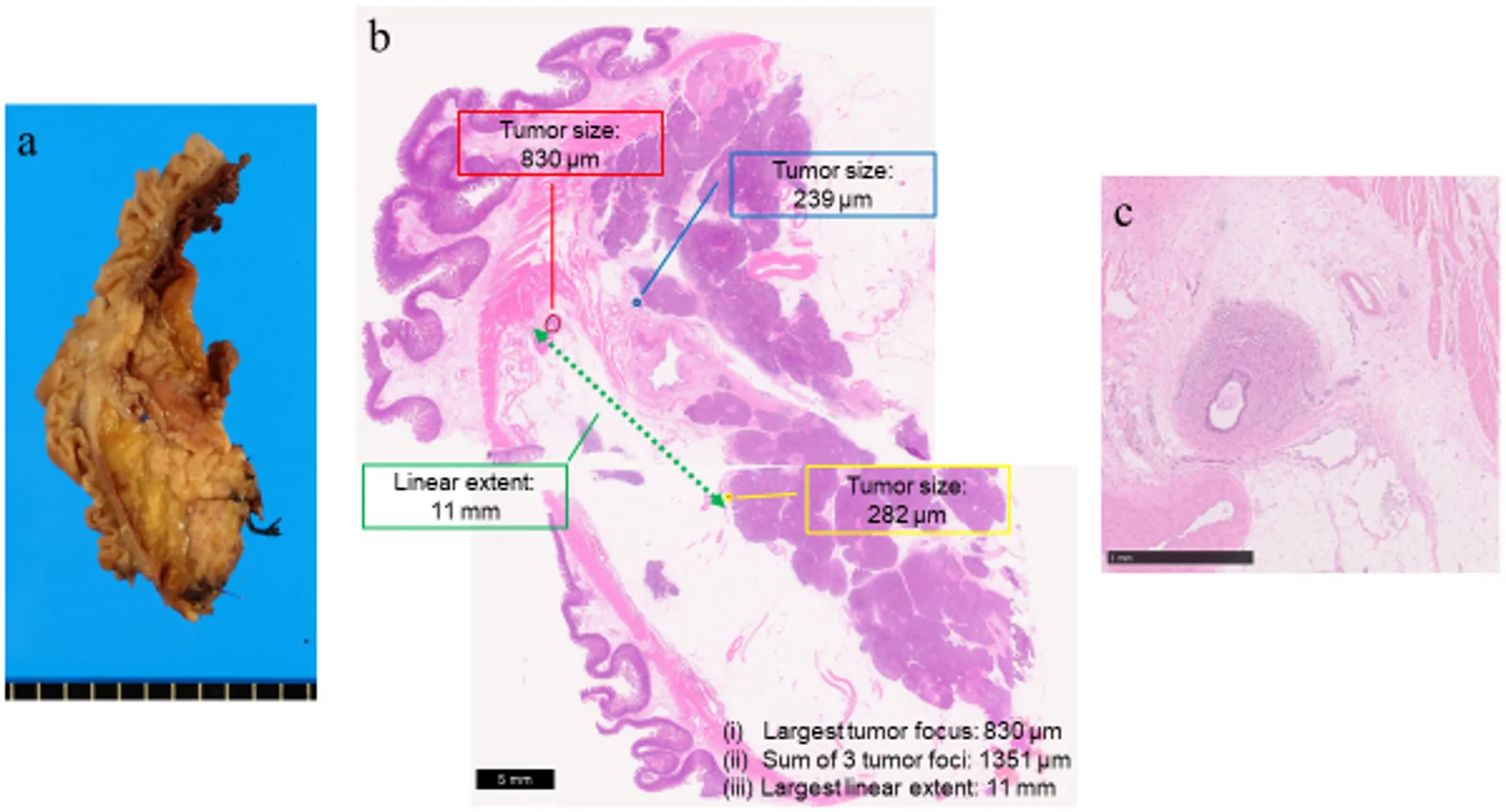
A representative case with a histopathological response grade 2 after preoperative chemotherapy. (**a)** Gross specimen (**b**) Mapped histological sections showing the distribution of residual tumor foci. Residual tumors are indicated by red, blue, and yellow circles. The maximum diameters of the red, blue, and yellow foci were 830, 239, and 282 μm. Depending on the measurement definition, tumor size was recorded as follows: (**i**) 830 μm when defined as the maximum diameter of the largest contiguous focus, (**ii**) 1,351 μm when defined as the sum of the maximum diameters of all foci, and (**iii**) 11 mm when defined as the maximum span including the intervening stroma between foci (green dotted arrow). (**c**) High-magnified view of the red area

Preoperative therapy increases stromal fibrosis, alters perfusion characteristics, and may reduce tumor-to-parenchyma contrast on CT [6, 29, 44, 45]. Because routine CT sizing is largely based on attenuation differences, residual tumors may become isoattenuating or ill-defined after therapy, making linear measurements vulnerable to underestimation [6, 10, 11, 29, 45]. Moreover, treated tumor beds may contain variable mixtures of fibrosis, inflammation, necrosis, and residual carcinoma, which cannot be reliably separated using conventional imaging [6, 29, 45, 46]. To assess the influence of preoperative therapy on the radiologic-pathologic correlation in tumor size assessment, Das et al. compared treatment-naïve and neoadjuvant-treated PDAC cohorts [38]. The correlation coefficient (r) between preoperative EUS-based tumor size and postoperative pathological tumor size decreased from 0.726 (*P* < 0.001) in 340 treatment-naïve PDACs to 0.214 (*P* < 0.001) in 365 neoadjuvant-treated PDACs [38]. In a separate cohort of 343 neoadjuvant-treated PDAC patients, Wei et al. reported that the CT-measured longest diameter after preoperative treatment correlated with pathologic tumor size (*r* = 0.72, *P* < 0.001), whereas this correlation was not observed in patients with ypT0 disease or pathologic tumor size ≤ 1.0 cm [47]. In addition to therapy-related changes in imaging appearance, the discontinuous distribution of viable tumor cells after preoperative therapy represents another important mechanism of measurement discordance. Figure 4 shows representative CT images, gross findings, and microscopic findings for each therapeutic effect grade according to the *General Rules for the Study of Pancreatic Cancer* of the Japanese Pancreas Society [5]. Pathologic evaluation of pancreatectomy specimens after preoperative therapy frequently demonstrates residual viable tumor cells persisting as isolated single cells or small nests scattered within a fibrotic tumor bed [48, 49]. To clarify how tumor size is defined and interpreted across major pathology protocols, the approaches to tumor size measurement, both in general and after preoperative therapy, are summarized in Table 2. Importantly, these protocols collectively acknowledge that post-therapy PDAC requires combined gross and microscopic assessments and may not be adequately captured by a single, simplistic size concept alone. According to the *General Rules for the Study of Pancreatic Cancer* (eighth edition) of the Japanese Pancreas Society, tumor size is defined as the maximum diameter, including both the noninvasive and invasive components. In cases with multiple lesions, the number, location, and maximum diameter of each lesion were documented. However, these rules do not provide explicit criteria for whether non-contiguous tumor foci should be regarded as separate multiple lesions or as invasive components discontinuous from the main tumor mass [5]. In addition, for tumor size assessment after preoperative chemotherapy, no explicit definition has been provided regarding the extent to which residual tumor foci that are not contiguous with the main residual lesion should be incorporated into the tumor size measurement. In contrast, international pathology protocols place greater emphasis on the microscopic validation of tumor extent after preoperative therapy. The Royal College of Pathologists of Australasia describes two pragmatic approaches when residual viable tumors are present in multiple foci: measuring the entire region bounded by viable tumor cells, including intervening fibrosis, as a single lesion or summing the largest dimension of each residual focus [50]. Similarly, both the College of American Pathologists (CAP) and the International Collaboration on Cancer Reporting state that the post-treatment pT (ypT) classification should be based on the extent of viable tumors, excluding the acellular mucin pools. These guidelines recognize microscopic residual disease and recommend considering the overall distribution of viable tumor foci when estimating post- and preoperative treatment tumor sizes. The CAP further notes that multiple viable foci within the same tumor mass may be added to determine the maximum linear dimension for staging [51–53]. Figure 5 illustrates the variation in tumor size estimates according to different definitions of tumor size measurement.

**Table 2:** Comparison of tumor size measurement methods among the major guidelines

Protocol	Definition of tumor size	Tumor size measurement after preoperative chemotherapy	Tumor response/regression assessment
*General Rules for the Study of Pancreatic Cancer* [5]	TS (JPS): maximum diameter of the primary tumor (gross measurement corroborated microscopically) JPS tumor category is defined by local extension; size-based tumor categories depend on the staging system used (e.g., AJCC/UICC TNM)	No specific method for incorporating non-contiguous residual foci is defined.	Assessed by the estimated residual cancer rate (proportion of viable cancer cells) Acknowledges difficulty in estimating pre-treatment tumor extent
Royal College of Pathologists of Australasia (RCPA) [50]	Gross measurement with microscopic confirmation Multifocal disease recorded; staging based on largest lesion	If multiple residual foci are present: (i) Measure entire viable tumor–bounded region. (ii) Sum the largest dimension of each focus. No consensus: The method should be specified.	Based on the extent and proportion of residual viable tumor
International Collaboration on Cancer Reporting (ICCR) [52]	Site and size of individual tumors recorded Staging based on the largest tumor, with microscopic validation	If multiple residual foci are present: (i) Measure the largest linear dimension of the entire area involved by viable residual tumor cells, including intervening non-cancerous tissue. (ii) Sum the largest dimension of each focus.	Based on the extent and proportion of residual viable tumor
College of American Pathologists (CAP) [53]	T1–T3 size based (gross measurement corroborated microscopically)	For ypT after therapy: use size/extent of viable tumor with combined gross + microscopic assessment. Acellular mucin pools may be present after chemoradiation and should not be interpreted as residual tumor. Multiple viable foci within the same tumor mass may be added to obtain the maximum linear dimension.	Based on the extent and proportion of residual viable tumor

TS: tumor size; JPS: Japanese Pancreas Society; AJCC: American Joint Committee on Cancer; UICC: Union for International Cancer Control; TNM: tumor–node–metastasis

Although tumor size was not directly compared according to each definition, Zhang et al. reported that the ypT stage based on the *AJCC Cancer Staging Manual* eighth edition, may vary according to the method used for tumor size measurement [54, 55]. When gross size with microscopic adjustment, which reflects the tumor bed including therapy-induced fibrosis, was used for staging, tumors were classified as T0/T1/T2/T3 in 9/35/42/20 cases, respectively [55]. In contrast, when the largest microscopic focus size, defined as the maximum dimension of a continuous viable tumor focus, was applied, the distribution shifted to 9/85/12/0, eliminating the T3 classification and reassigning many tumors to lower ypT categories [55]. Therefore, after effective preoperative therapy, radiologically measurable abnormalities do not necessarily correspond to viable tumor cells, and discontinuous microscopic residual disease complicates pathological tumor size assessment, resulting in radiologic–pathologic measurement discrepancies [49, 51–53, 55].

### Treatment-specific effects of preoperative therapy

The magnitude and nature of this discrepancy can vary according to the type of preoperative therapy. Comparative clinical data indicate that radiographic–pathologic discordance patterns differ between preoperative chemotherapy and preoperative chemoradiotherapy, particularly in scenarios where radiographic changes are minimal despite the histologic response [56]. In general, radiation-containing regimens are strongly associated with dense fibrosis and stromal remodeling [44–46, 56], which may further impair margin definition on images and promote underestimation of tumor extent at the infiltrative rim [6, 29, 45].

It remains unclear whether the degree of radiologic–pathologic tumor size discrepancy varies according to the chemotherapy regimen. Available comparative studies have focused mainly on radiographic response, pathologic response, and survival, rather than on the measurement error itself [57, 58]. In a multicenter cohort study, Macedo et al. reported no significant difference in the pathologic response distribution between the neoadjuvant fluorouracil, leucovorin, irinotecan, and oxaliplatin (FOLFIRINOX) and gemcitabine plus nab-paclitaxel (GnP) groups [57]. Conversely, Wolfe et al. reported that neoadjuvant modified FOLFIRINOX was associated with higher partial or complete response rates than GnP (16/52 vs. 1/20, *P* = 0.028) based on Response Evaluation Criteria in Solid Tumors (RECIST), with greater mean radiographic tumor size reduction (17% vs. 12%), smaller pathologic tumor size (2.34 vs. 3.16 cm, *P* = 0.03), and more favorable treatment response grades [58].

Therefore, post-treatment tumor size should be interpreted in light of both the use of radiotherapy and the systemic regimen administered, although direct evidence linking specific regimens to the extent of tumor size measurement errors remains limited.

### Limitations of size-based response assessment

Size-based response metrics for pancreatic cancer have inherent structural limitations. Conventional response frameworks, such as RECIST version 1.1 [7], focus on changes in the size of measurable lesions on imaging. In contrast, pathologic treatment-related changes often occur as reductions in tumor cellularity and replacement by stroma, such that biologically meaningful therapeutic responses cannot be adequately captured by changes in tumor size alone [7, 46, 49, 55]. A large clinical series similarly showed that major pathologic responses can occur with only modest radiographic size changes, thus underscoring the weak coupling between post-therapy size and residual viable tumor burden [46, 48, 56]. Taken together, preoperative therapy may promote fibrosis and scattered residual disease, thereby accentuating the tendency of CT-based tumor size measurements to underestimate the true pathological extent of the disease. Accordingly, in contemporary PDAC management, it is important to recognize the inherent limitations of response assessment based solely on tumor size [6, 9–11, 29, 33, 44–46, 48–50, 59].

## Prognostic implications of tumor size: imaging vs. pathology

### Prognostic significance of imaging-based tumor size

Tumor size measured on preoperative imaging, particularly contrast-enhanced CT, has long been used as a surrogate marker of the tumor burden and prognosis in patients with PDAC. In surgery-first cohorts, a larger CT-measured tumor size has been consistently associated with inferior OS, thus supporting its role as a pragmatic preoperative risk-stratification parameter [8, 9, 17, 60]. Specifically, in upfront-surgery patients, a preoperative CT tumor size > 2 cm was independently associated with a significantly increased risk of death (hazard ratio [HR] = 2.30, 95% confidence interval [CI] = 1.19–4.42) compared with tumors ≤ 2 cm [60].

However, the prognostic value of imaging-based tumor size appears to be context-dependent and is substantially attenuated during preoperative treatment. In the same cohort, no significant association between preoperative CT-measured tumor size and OS was observed among patients who received preoperative therapy (HR = 0.84, 95% CI, 0.43–1.64; *P* = 0.61) [60]. These findings indicate that attenuation-based size measurements may lose their ability to reflect a clinically meaningful residual disease burden after preoperative treatment.

### Prognostic significance of pathologic tumor size

The pathological tumor size is a central component of current tumor classification systems and it remains one of the most consistently validated prognostic factors for resected pancreatic cancer. A systematic review and meta-analysis of 28 observational studies, including 23,945 resected PDAC patients, without specific stratification according to preoperative therapy, demonstrated that tumors > 2 cm were associated with a worse OS by a multivariate analysis (HR = 1.61; 95% CI, 1.35–1.91; *P* < 0.0001) [8].

In the post- and preoperative treatment settings, size-based ypT classification provides improved prognostic stratification compared with earlier staging systems. In a large cohort of preoperatively treated pancreatic cancer patients, the application of the size-based ypT categories of the *AJCC Cancer Staging Manual* 8th edition demonstrated clear survival separation, with ypT3 tumors associated with significantly worse OS than ypT1–ypT2 tumors (HR ≈ 2.0) [61].

The prognostic performance of pathological size-based staging after preoperative therapy may also depend on the definition of residual tumor size. Zhang et al. compared ypT staging based on gross size with microscopic adjustment (GS), reflecting the gross tumor bed with microscopic confirmation, and the largest microscopic focus size (MFS), defined as the maximum dimension of a continuous viable tumor focus [55]. Although these definitions do not correspond exactly to any single protocol summarized in Table 2, GS broadly reflects gross measurements with microscopic confirmation, whereas MFS reflects the largest contiguous viable tumor focus. In multivariate analysis, MFS-based ypT stage was an independent predictor of both disease-free survival (DFS) and OS (DFS: HR = 3.83; 95% CI, 1.67–8.79; *P* = 0.002; OS: HR = 4.86; 95% CI, 1.97–12.02; *P* < 0.001) [55]. In contrast, GS-based ypT stage was associated with OS but not with DFS (OS: HR, 1.64; 95% CI, 1.06–2.55; *P* = 0.027; DFS: HR, 1.36; 95% CI, 0.93–1.99; *P* = 0.115) [55]. These findings suggest that measuring pathological tumor size after preoperative therapy based on the residual viable tumor burden or broader fibrotic tumor bed may be appropriate.

### Prognostic implications of radiologic–pathologic size discrepancy

Beyond absolute tumor size, the discordance between imaging-based and pathological tumor measurements may provide prognostic information. A previous study comparing preoperative EUS with postoperative pathology demonstrated that patients with a substantial reduction in tumor size from imaging to pathology experienced significantly improved survival after preoperative therapy [38]. In that analysis, a ≥ 47% decrease in tumor size from EUS to pathology was independently associated with prolonged OS, suggesting that marked size reduction may reflect the effectiveness of preoperative therapy rather than imaging inaccuracy alone [38].

Conversely, direct evidence that CT-based underestimation of primary tumor size independently worsens survival is limited because most radiologic–pathologic correlation studies have not been designed for T-factor-specific outcome analyses. Although a National Cancer Database analysis of clinical stage I pancreatic adenocarcinoma showed that pathologic upstaging was associated with worse survival, this upstaging reflected both T- and N-factor underestimation rather than tumor size understaging alone [62]. Therefore, the radiologic underestimation of tumor size independently predicts postoperative prognosis and remains an important subject for future investigation.

## Future directions beyond tumor size

### Functional imaging

Experience with other gastrointestinal malignancies has highlighted that treatment response and prognosis are not determined by size changes alone. In colorectal liver metastases treated with modern chemotherapy, CT morphological response correlated with pathological response and survival, thus providing clinically useful, non-size-based risk stratification beyond the RECIST diameter change [63, 64]. These data support the broader concept that phenotypic changes on imaging may better capture biologically meaningful responses than linear size, particularly when therapy induces fibrosis or alters enhancement without proportional shrinkage of the tumor.

In PDAC, metabolic response assessment using fluorine-18 fluorodeoxyglucose positron emission tomography with CT (^18^F-FDG PET/CT) has been explored as a complementary functional imaging modality after preoperative therapy. Reductions in metabolic tumor parameters, including maximum standardized uptake value (SUVmax), peak standardized uptake value (SUVpeak), and metabolic tumor volume on ^18^F-FDG PET/CT following preoperative chemotherapy, have been associated with an improved OS and recurrence-free survival, indicating that the metabolic response may provide prognostically relevant information beyond anatomic diameter change [65–67]. Additionally, in patients with borderline resectable or locally advanced disease, changes in positron emission tomography/MRI metabolic metrics correlated with major pathologic responses and survival outcomes, further supporting the value of metabolic imaging biomarkers in evaluating treatment efficacy [68]. However, metabolic assessment using ^18^F-FDG PET/CT is susceptible to systemic factors. In particular, diabetes mellitus and hyperglycemia may alter the FDG uptake, complicating the interpretation of treatment-related changes in SUV metrics. Furthermore, because relatively low tumor cellularity is observed in PDAC owing to the characteristically abundant desmoplastic stroma [16, 69], the low SUVpeak of the lesions does not directly reflect the volume of residual viable cancer cells after preoperative therapy [70, 71].

### Radiomics, artificial intelligence and advanced quantitative analysis

Radiomics aims to extract high-dimensional quantitative imaging features that may reflect intratumoral heterogeneity and microenvironmental composition not captured by a single tumor diameter [72]. In pancreatic cancer, CT radiomics has been linked to biological correlates such as genotype and stromal content, and delta radiomics has been explored to predict treatment response using machine-learning pipelines [73, 74].

Evidence that artificial intelligence (AI) or radiomics directly improves the accuracy of tumor size measurement remains limited; however, advanced quantitative image analysis has been evaluated for this purpose in recent studies. Michallek et al. compared the accuracy of size assessment between conventional CT, CT-derived fractal dimension maps, multiparametric MRI, and gross pathology in PDAC [59]. Conventional CT underestimated tumor diameter by 5.7 mm compared with gross pathology, whereas fractal dimension maps and multi-parametric MRI showed smaller differences from pathology, at 0.6 mm and − 0.9 mm, respectively [59]. In a validation cohort using multiparametric MRI as the standard reference, CT underestimated the tumor diameter by 10.6 mm, whereas fractal dimension maps showed closer agreement, with a difference of 1.5 mm [59]. These findings suggest that advanced quantitative image analysis may reduce CT-based size underestimation by improving the visualization of the tumor rim. Nevertheless, whether AI, radiomics, or related quantitative approaches can reliably reduce radiologic–pathologic discrepancies after preoperative therapy remains an issue [72–75].

### Pathologic standardization after preoperative treatment

After preoperative treatment, gross tumor borders are often indistinct, and viable carcinoma may persist as scattered foci within a fibrotic bed, thus making tumor size assignment and ypT categorization vulnerable to sampling, mapping, and definitional variabilities. Although existing consensus statements and guidelines emphasize standardized specimen handling and reporting in treated PDAC, tumor size measurement in the setting of discontinuous residual disease remains an unresolved issue, as discussed above. To enhance comparability across studies and registries, clearer operational definitions that distinguish “tumor extent” from the “largest contiguous viable tumor focus” are required [52, 53, 55, 76].

## 6. Conclusions

In the era of preoperative therapy for PDAC, clinical tumor staging based solely on tumor size assessment has inherent limitations. Moreover, the discordance between radiologic and pathologic tumor sizes may reflect not only differences in measurement methodology, but also the underlying tumor biology. Future efforts should explore objective and reproducible surrogate markers beyond tumor size and consider their integration into clinical staging frameworks.
